# The Predictive Processing Paradigm Has Roots in Kant

**DOI:** 10.3389/fnsys.2016.00079

**Published:** 2016-10-10

**Authors:** Link R. Swanson

**Affiliations:** ^1^Department of Philosophy, University of MinnesotaMinneapolis, MN, USA; ^2^Center for Cognitive Sciences, University of MinnesotaMinneapolis, MN, USA; ^3^Minnesota Center for Philosophy of Science, University of MinnesotaMinneapolis, MN, USA

**Keywords:** predictive processing, Kant, top-down processing, hyperpriors, generative model, schemata, analysis by synthesis, imagination in perception

## Abstract

Predictive processing (PP) is a paradigm in computational and cognitive neuroscience that has recently attracted significant attention across domains, including psychology, robotics, artificial intelligence and philosophy. It is often regarded as a fresh and possibly revolutionary paradigm shift, yet a handful of authors have remarked that aspects of PP seem reminiscent of the work of 18th century philosopher Immanuel Kant. To date there have not been any substantive discussions of how exactly PP links back to Kant. In this article, I argue that several core aspects of PP were anticipated by Kant (1996/1787) in his works on perception and cognition. Themes from Kant active in PP include: (1) the emphasis on “top-down” generation of percepts; (2) the role of “hyperpriors”; (3) the general function of “generative models”; (4) the process of “analysis-by-synthesis”; and (5) the crucial role of imagination in perception. In addition to these, I also point out that PP echoes Kant’s general project in that it aims to explain how minds track causal structure in the world using only sensory data, and that it uses a reverse-engineer or “top-down” method of analysis. I then locate a possible source of Kant’s influence on PP by tracing the paradigm back to Hermann von Helmholtz, who saw himself as providing a scientific implementation of Kant’s conclusions. I conclude by arguing that PP should not be regarded as a new paradigm, but is more appropriately understood as the latest incarnation of an approach to perception and cognition initiated by Kant and refined by Helmholtz.

## Introduction

“Let’s put it this way: Kant knew nothing about the platypus, and that should not worry us, but if the platypus is to solve its own identity crisis, it ought to know something about Kant.”

— Umberto Eco (2000), *Kant and the Platypus*.

Predictive processing (PP) is a paradigm in computational and cognitive neuroscience proposing that “perception involves the use of a unified body of acquired knowledge (a multi-level “generative model”) to predict the incoming sensory barrage” (Clark, 2015c, p. 5). PP has recently attracted significant attention across domains, including psychology, linguistics, robotics, artificial intelligence and philosophy (see Andy Clark’s BBS target article (Clark, 2013) with dozens of responses, as well as the Frontiers Research Topic (Frontiers in Theoretical and Philosophical Psychology, 2013)). PP combines and builds upon previous ideas about the role of “unconscious inference” in perception (Helmholtz, 1925; Barlow, 1961; Gregory, 1970), the process of “analysis by synthesis” in psychology (Neisser, 1967), the “predictive coding” approach in neuroscience (Srinivasan et al., 1982; Rao and Ballard, 1999; Huang and Rao, 2011) and “generative models” and related probabilistic computational principles (MacKay, 1956; Mumford, 1992; Dayan et al., 1995; Hinton, 2007a,b; Tenenbaum et al., 2011). By integrating all of these approaches into a unified, hierarchical, probabilistic model of brain function, the PP paradigm promises to offer “a computationally tractable version of the so-called Bayesian Brain Hypothesis” (Clark, 2013, p. 191)^1^. In this article I focus on recent “big picture” formulations of PP that attempt to offer a unified theory of brain function, exemplified by the work of Friston (2003, 2005); Friston et al. (2006), as recently outlined by philosophers Clark (2013) and Hohwy (2013).

While opinions differ concerning the “implied vision of mind” contained within the PP paradigm (Clark, 2015c, p. 3), several authors have made quick remarks stating that certain aspects of PP seem reminiscent of 18th century philosopher Immanuel Kant’s work on cognition and perception. For example, Clark (2013, p. 196) remarks that at certain points PP can evoke an “almost Kantian feel,” and Gładziejewski (2016, p. 16) states that PP “presents us with a view of perception as Kantian in spirit.” PP literature has been cited in support of the argument that recent cognitive science is outlining a “Kantian brain” (Fazelpour and Thompson, 2015). The PP paradigm has even been labeled (pejoratively) as a “neo-neo-Kantian view of the relationship between mind and world” (Anderson and Chemero, 2013, p. 204). Hohwy even notes that “there is certainly a distinct Kantian element” to the PP paradigm, but goes on to merely mention a few of Kant’s ideas in a list before truncating the list with “etc.” (Hohwy, 2013, p. 5).

With this article I hope to contribute a closer examination of the relationship between the PP paradigm and the ideas of Kant. I pick up where Hohwy left off with “etc.” by presenting and defending clear links between specific elements of PP and key ideas proposed by Kant. First, I point out that Kant was an early pioneer of the “top-down” *analytical method* common to PP theorists, and that, like PP, he used this analytical method to investigate how minds can track causal structure. Next, I present five *distinct components* of PP that have significant links with five key ideas proposed by Kant. Finally, I argue that the similarities between Kant and PP should not come as a surprise if we consider the fact that PP is historically connected to Kant through Hermann von Helmholtz.

## Reverse-Engineering, Top-Down Analysis and Kant’s Transcendental Method of Argument

In the field of electrical engineering, reverse-engineering is defined as “the act of creating a set of specifications for a piece of hardware by someone other than the original designers, primarily based upon analyzing and dimensioning a specimen or collection of specimens” (Rekoff, 1985, p. 244). To reverse-engineer a system is to start with the complete functioning system and apply a *functional* analysis from the “top-down” in an effort to discover how its parts achieve its overall function. Cognitive scientists commonly attempt to reverse-engineer the mind—a method often termed “top-down analysis”—by observing the fully functioning perceptual-cognitive system in an attempt to discover the necessary components that *must* be required for such a system to operate in the way that it does (Pinker, 1999; Griffiths et al., 2010; Tenenbaum et al., 2011). Contrast this with the forward engineering approach, which for cognitive science means a “bottom-up approach, beginning with a characterization of neural mechanisms and exploring what macro-level functional phenomena might emerge” (Griffiths et al., 2010, p. 357). While theorists working within the PP paradigm commonly use both bottom-up and top-down methods of analysis, use of the top-down “reverse-engineer the mind” approach is characteristic of PP’s overall analytical methodology (Hohwy, 2013).

Kant adopted a top-down analytical approach as a central guiding principle, known as the “transcendental method of argument” (Kant, 1996/1787; Kitcher, 1996; Brook, 2007; Stern, 2015). A transcendental argument justifies some concept or claim by showing that it is a necessary condition on the possibility of some other fact of experience (Stroud, 1968; Stern, 2015). Kant is recognized as the first in Western philosophy to fully leverage transcendental arguments, and this is often cited as a defining characteristic of what sets Kant’s analytical methodology apart from that of his contemporaries (Kitcher, 1996; Brook, 2007; Stern, 2015). Kitcher (1996) points out that Kant *pioneered* what is now being called the top-down approach in cognitive science. “In contemporary terminology, where much current research is descriptive and “bottom-up”, Kant’s approach was “top-down”. He tried to analyze the sorts of processes that were necessary for genuine cognition to be possible” (Kitcher, 1996, p. xliv). Recently, Griffiths et al. (2010, p. 357) call for a top-down analytical approach to studying perception and cognition, stating that “cognitive science aims to reverse-engineer the mind… a top-down analysis of cognition starting with the function of cognitive processes… yields greater flexibility for exploring the representations and inductive biases that underlie human cognition”. Kant made a similar call in his 1783 *Prolegomena to Any Future Metaphysics That Will Be Able to Come Forward as a Science*: “We will start from the position that… cognition is actual; but we must nonetheless next investigate the ground of this possibility, and ask: how this cognition is possible… ” (Kant, 1783, sec. 4:276). Brook (2007) argues that Kant should be recognized as the “grandfather of cognitive science” in part because he pioneered the application of this style of top-down analysis to the study of cognition and perception. This fact supports the case that PP has roots in Kant, since some of the strongest advocates of the top-down analytical approach in cognitive science are those working on PP theories (Hohwy, 2013).

The “top-down” or “reverse-engineer” method of analysis—a defining characteristic of the analytical methodology of probabilistic approaches to cognition (and PP in particular)—was pioneered by Kant and central to his philosophical method. However, top-down analysis cannot get off the ground without a clearly defined functional specimen to serve as the “top” for the reverse-engineering process. In the next section I argue that Kant and PP both define the primary function of cognition and perception as the ability to track causal structure without direct access to real-world causes.

## How Can Minds Track Causal Structure?

For Bayesian models of cognition and perception in general, “the big question is this: how does the human mind go beyond the data of experience?” (Griffiths et al., 2008, p. 59). In such models, including PP theories, the *causes* of sensations are commonly referred to as “hidden causes” or “distal causes” (Rao and Ballard, 1999; Feldman and Friston, 2010; Battaglia et al., 2012; Clark, 2013; Hohwy, 2013; Purves et al., 2015). They are *hidden* because the only “data” that brains have to work with are the *effects* of stimulated sense organs. “In biological perception, the brain directly measures sensory cues but does not directly measure external world properties” (Battaglia et al., 2012). The PP paradigm is ultimately aimed at explaining how brains can track real-world causes using only sensory effects (Körding et al., 2007; Clark, 2013; Hohwy, 2013; Purves et al., 2015). “The problem of perception is the problem of using the effects—that is, the sensory data that is all the brain has access to—to figure out the causes” (Hohwy, 2013, p. 13). Clark gives a similar characterization. “For, the task of the brain, when viewed from a certain distance, can seem impossible: it must discover information about the likely causes of impinging signals without any form of direct access to their source” (Clark, 2013, p. 183). This position is what Hohwy (2013) terms “the skull-bound brain” and what Clark (2013, p. 183) characterizes as the “view from inside the black box”.

The PP paradigm thus aims to provide a neurally plausible set of mechanisms by which brains accomplish causal inference and overcome the challenges of induction (Friston, 2003; Hohwy, 2013). Induction itself has enjoyed a central role in the history of science, philosophy and philosophy of science. “David Hume is a pivotal character in this regard” (Hohwy, 2013, p. 6). Hume developed arguments that challenged the existence of necessary causal connections between sensations (Hume, 1739, bk. I, part III, section vi). The PP paradigm has been framed as an answer to Hume’s challenge in that it aims to offer an account for how causal structure is extracted from statistical regularities that occur in sensory stimulation (Hohwy, 2012, 2013; Dennett, 2013; Flores, 2015). PP’s answer to Humean problems of induction rests on proposed neural computations based on Bayesian principles (Knill and Pouget, 2004; Blokpoel et al., 2012; Hohwy, 2013; Clark, 2015a; Mikowski, 2016). Interestingly, Bayesian principles themselves arose in part as a response to Hume’s problem of induction (Gillies, 1987; McGrayne, 2011). Thomas Bayes’ ideas on probability were not published until after his death when his friend Richard Price presented an essay to the Royal Society of London, which included Bayes’ ideas along with some important additions by Price (Bayes and Price, 1763; Gillies, 1987; McGrayne, 2011). At first glance, Price did not think much of Bayes’ essay on the probability of causes. However, “once Price decided Bayes’ essay was the answer to Hume’s attack on causation, he began preparing it for publication” (McGrayne, 2011, pp. 10–11; see also Gillies, 1987, p. 325). Thus, without Price’s Hume-driven motivations, Bayes’ ideas would probably not have been published.

Contemporaneous with the Bayes/Price effort to respond to Hume, a quite different but no less influential response was in the works—from Kant. Kant advanced a unique, elaborate, and massively influential answer to Hume (Guyer, 2008). Kant’s “critical period”, during which he developed his most important work, began as a direct and explicit response to Hume’s challenge (Kant, 1783; Hatfield, 2006; Guyer, 2008). Kant famously recounts that it was Hume, who “first interrupted my dogmatic slumber and gave a completely different direction to my researches” (Kant, 1783, sec. 4:260). Kant goes on to explain that, once he finally embraced Hume’s challenge and was led to the conclusion that causal connections must have their origin in *minds* and not in real-world properties, he was driven to inquire what *other* aspects of experience might arise in this way, and that it was this initial inquiry that eventually led him to the conclusions of his transcendental idealism (Kant, 1783, sec. 4:260). Indeed, the problem of causation that Hume raised was so central to Kant’s project that Kant did not hesitate to characterize his entire *Critique of Pure Reason* simply as the “elaboration of the Humean problem in its greatest possible amplification” (Kant, 1783, sec. 4:261).

Throughout his critical period work, Kant maintained a sharp epistemic divide between sensory experiences—“appearances”—and the actual causes of sensations—“things in themselves” (Kant, 1996/1787; Allison, 2004; Stang, 2016). “What may be the case regarding objects in themselves and apart from… our sensibility remains to us entirely unknown. All we know is the way in which we perceive them.” (Kant, 1996/1787, sec. A42). This thesis, which Kant dubbed “transcendental idealism”, has generated much interpretive debate and controversy (Allison, 2004; Rohlf, 2016). Paton et al. (2013, p. 222) insist that, although some authors might downplay it, PP “does convey a somehow indirect mind-world relation”. This is presumably what Anderson and Chemero (2013, p. 204) refer to when they label PP as a “neo-neo-Kantian view of the relationship between mind and world”.

Thus, Kant and PP each aim to offer detailed accounts of how minds track “hidden causes” using only the data from the senses, and they both develop these accounts using methods of top-down analysis in an attempt to reverse-engineer perception and cognition. How do their accounts compare? In what follows I argue that if we compare some of the major theoretical postulates that Kant and PP each propose, we find that their kinship runs deeper than the general similarities outlined so far.

In the following sections I present five specific theoretical components of PP and show how each is connected to one of five specific key ideas proposed by Kant: (1) PP’s advocacy for a *reversal* of the traditional picture of perception is linked to Kant’s self-described “Copernican revolution”; (2) PP’s notion of *hyperpriors* is linked to Kant’s idea of “forms of appearances”; (3) PP’s principles of *generative models* are linked to Kant’s concept of “schemata”; (4) PP’s *analysis-by-synthesis* is linked to Kant’s proposal that analysis proceeds by synthesis; and (5) PP’s claim that imagination is required for perception is linked to Kant’s claim that imagination is required for perception.

## PP’s “Reversal” Is Kant’s “Copernican Revolution”

When considered within the short history of today’s cognitive science, PP offers a radical and revisionary stance on the relationship between percepts and external objects. A common strategy that many authors use for explaining this distinctive difference is to contrast PP with “standard,” “classical” or “traditional” approaches to perception and cognition (Engel et al., 2001; Lee and Mumford, 2003; Yuille and Kersten, 2006; Hohwy, 2013; Clark, 2015a,b). PP literature often describes “traditional” approaches as those that assume that perception is a *passive* process by which features of objects in the environment are *detected* by the sense organs and encoded into the nervous system and assembled in a bottom-up fashion. PP rejects this bottom-up conception of perception. “PP turns a traditional picture of perception *on its head*” (Clark, 2015a, p. 51; emphasis mine). PP urges that psychology and neuroscience would make better progress on the problems of perception if they would instead assume that brains actively *generate* percepts in a top-down manner, not by accumulating and combining input signals, but rather, by* issuing predictions or accounts of the current state of the input signals* based on hierarchical generative models that rely on prior probabilities and likelihood estimates (Kersten et al., 2004; Friston, 2005; Hohwy, 2013; Clark, 2015a). As repeatedly emphasized by many authors, this is not just a small modification of traditional accounts of perception.

In fact, it *profoundly reverses* how we conceive our relation to the world through the senses. A standard conception is that the senses convey a rich signal that somehow represents a worldly state of affairs, which the brain is passively soaking up in a bottom-up manner. On the [PP] view, this picture is reversed. The rich representation of worldly states of affairs is signalled in the top-down predictions of sensory input, maintained by the perceptual hierarchy in the brain (Hohwy, 2013, p. 47; emphasis mine).

On the PP account, the cortical hierarchy is constantly generating predictions from the top down that attempt to account for the causes of the bottom-up sensory stimulation (Friston, 2005). “This means that perceptual content *is* the predictions of the currently best hypothesis about the world” (Hohwy, 2013, p. 48). Hohwy (2013, p. 2) captures the overall upshot of this reversal of standard thinking about perception with a catchy slogan. “The sensory input to the brain does not shape perception directly: sensory input is better and more perplexingly characterized as feedback to the queries issued by the brain”. Keep this slogan in mind as we consider another famous slogan—from Kant—in what follows.

In the introduction to his *Critique of Pure Reason*, Kant urges that if we are to make any progress on understanding the relation between perception, cognition and external objects, we need a fundamental shift in thinking. Kant introduces his proposal by contrasting it with the “traditional” accounts of his time (recall from above that this rhetorical strategy is used by PP theorists when describing their accounts). “Thus far it has been assumed that all our cognition must conform to objects” (Kant, 1996/1787, sec. B xvi). Here Kant is referring to the theories of thinkers that came before him, which, he argues, “have come to nothing,” because they assume that sense organs passively receive impressions stamped by external objects (Kant, 1996/1787, sec. B xvi). “Let us, therefore,” Kant proposes, “try and find out by experiment whether we shall not make better progress on the problems of metaphysics if we assume that *objects must conform to our cognition*” (Kant, 1996/1787, sec. B xvi; emphasis mine). By *reversing* our assumptions about the relation between cognition and the objects of external perception, Kant argues that we will be in a better position to understand how the perceptual-cognitive system can possibly be as we experience it. Kant claims that this position enables us to discover the properties of the cognition that is being reverse-engineered, “i.e., a cognition that is to ascertain something about (objects) before they are given to us” (Kant, 1996/1787, sec. B xvi). Kant considered this reversal to be so crucial to our investigations of perception and cognition that he immodestly claimed that it would deliver results as monumental as the ideas of Copernicus were for astronomy.

The situation here is the same as was that of Copernicus when he first thought of explaining the motions of celestial bodies. Having found it difficult to make progress there when he assumed that the entire host of stars revolved around the spectator, he tried to find out by experiment whether he might not be more successful if he had the spectator revolve and the stars remain at rest. Now, we can try a similar experiment in metaphysics, with regard to our intuition of objects. If our intuition had to conform to the character of its objects, then I do not see how we could know anything* a priori* about that character. But I can quite readily conceive of this possibility if the object (as object of the senses) conforms to the character of our power of intuition (Kant, 1996/1787, sec. B xvii).

At this point, it should be clear that Kant’s call for a “Copernican” reversal of the traditional assumptions about perception anticipates the PP paradigm in important ways. PP “profoundly reverses” (Hohwy, 2013, p. 47) traditional assumptions about perception with its premise that “the world only tells us things in the sense that it provides answers to the questions we pose of it” (Hohwy, 2013, p. 225). Kant clearly anticipates this when he advocates that we should “assume that objects must conform to our cognition” (Kant, 1996/1787, sec. B xvi). Kant’s slogan is echoed over 200 years later in present-day language with Hohwy’s slogan that sensory input is best conceived “as feedback to the queries issued by the brain” (Hohwy, 2013, p. 2). Kant argued that his proposed shift in thinking would herald a new era in our understanding of perception and cognition. If a PP paradigm shapes up to be the revolutionary shift that many fancy it to be (Clark, 2013; Dennett, 2013; Hohwy, 2013; Madary, 2015; Purves et al., 2015), then Kant’s “Copernican revolution” might finally be catching on.

The “Copernican reversal” alone might prompt us to nominate Kant as the early forefather of the PP paradigm, at least with respect to this shared fundamental premise. Yet in the following sections I present evidence that Kant proposed even more specific theoretical ideas about perception and cognition that are now emerging as scientific hypotheses within the PP paradigm.

## Hyperpriors and Kant’s “Forms of Appearance”

As explained in the previous section, at the core of PP is the proposal that the fundamental mechanisms of perception involve something akin to (mostly unconscious) predictions, and that percepts essentially *are* these predictions. To arrive at predictions, brains require something on which these predictions can be based—predictive systems require *constraints* on the set of prior probabilities and likelihoods that should be taken into account as they finalize and settle upon a set of predictions for any given sensory-neural situation (Friston, 2003; Kemp et al., 2007; Tenenbaum et al., 2011; Blokpoel et al., 2012; Clark, 2013; Hohwy, 2013). Without such constraints, it is impossible for any intelligent system to narrow down the possibilities enough to settle on a single hypothesis or set of hypotheses (Russell and Norvig, 2010; Tenenbaum et al., 2011; Blokpoel et al., 2012). Linguists and developmental psychologists tend to refer to these cognitive mechanisms as “constraints”, while machine learning and artificial intelligence researchers tend to use the term “inductive biases” (Tenenbaum et al., 2011). In PP and Bayesian statistics literature, these probabilistic constraints are known as “priors” (Tenenbaum et al., 2011; Clark, 2013; Hohwy, 2013). While priors allow inductive systems to select a single hypothesis from a larger set of possible hypotheses (know as a “hypothesis space”), machine learning researchers have discovered that in order to achieve the complex representational abilities found in human cognition—from children to scientists—a *hierarchical system* of priors is required (Kemp et al., 2007; Tenenbaum et al., 2011; Clark, 2013). The key idea is that some priors in the hierarchy are more *abstract*, so the system can leverage “not just a single level of hypotheses to explain the data but multiple levels: hypothesis spaces of hypothesis spaces, with priors on priors” (Tenenbaum et al., 2011, p. 1282). Priors that are more abstract and fundamental, the rest are often called “hyperpriors” (see Hohwy et al., 2008; Clark, 2013; Friston et al., 2013) or “overhypotheses” (Goodman, 1983; Kemp et al., 2007). A multilayered, bidirectional, recursive process of hypothesis generation is a requirement addressed by hierarchical predictive coding models of brain function, and hyperpriors are crucial to such models (Friston and Kiebel, 2009; Blokpoel et al., 2012).

Prime examples of hyperpriors in the predictive perceptual system include the brute constraints imposed by *space* and *time*—e.g., “that there is only one object (one cause of sensory input) in one place, at a given scale, at a given moment,” or the fact that “we can only perform one action at a time, choosing the left turn or the right but never both at once” (Clark, 2013, p. 196). Abstract internal knowledge of space and time—spatial and temporal hyperpriors—are thought to narrow and restrict large swaths of possible hypothesis spaces, thereby aiding the formation of decisive perceptual predictions regarding the external objects causing incoming stimuli (Clark, 2013). This narrowing of possible hypotheses is critical to the entire probabilistic inference process—without it the required Bayesian computations become intractable (Tenenbaum et al., 2011; Blokpoel et al., 2012; Clark, 2013; Kwisthout, 2014). Spatial and temporal hyperpriors can thus be usefully conceived of as *necessary conditions* on the possibility of probabilistic perceptions of external objects. Keep this in mind during the following discussion of Kant’s account of the nature of space and time.

Clark explicitly mentions Kant during a discussion of hyperpriors. “Hyperpriors are essentially “priors upon priors” embodying systemic expectations concerning very abstract (at times almost “Kantian”) features of the world” (Clark, 2015a, p. 174). Here is a rare instance in the PP literature where Kant is invoked by name. But what *exactly* did Kant say that fits this description of hyperpriors?^2^ In the section of *Critique of Pure Reason* known as the “Transcendental Aesthetic”, Kant firmly distinguishes the “matter” of sensation from the “form” of sense experience (for an excellent overview see Hatfield, 2006). “Whatever in an appearance corresponds to sensation I call its *matter*; but whatever in an appearance brings about the fact that the manifold of the appearance can be ordered in certain relations I call the *form* of appearance” (Kant, 1996/1787, sec. A 20). Kant identifies two primal “forms” that shape the “matter” of sensation—namely, *space* and *time*. Importantly, Kant insists that spatial and temporal properties are endogenous features of cognition that impose *formal constraints* on the possibility of any experience of outer objects (Kant, 1996/1787; secs. B33–73; Hatfield, 2006). In other words, they are principles of cognition which enable the experience of outer objects. “Space is an *a priori* presentation that necessarily underlies outer appearances.” (Kant, 1996/1787, sec. B39).

Kant’s proposal that space and time are features of cognition that *form, constrain and restrict* possible perceptions of outer objects is echoed in explanations of the role of hyperpriors in PP accounts of perception. Without spatial and temporal hyperpriors, the objects of perception that putatively result from PP would be impossible (Tenenbaum et al., 2011; Blokpoel et al., 2012). This is much like Kant’s position that space and time are features of cognition that constrain the possibility of the experience of outer objects, and may be similar to Clark’s description of hyperpriors as evoking “an almost “Kantian” feel” (Clark, 2013, p. 196). Clark even echoes Kant’s use of the word *formal*. “The use of such a representational *form* would amount to the deployment of an implicit *formal* hyperprior (*formal*, because it concerns the *form* of the probabilistic representation itself)…” (Clark, 2013, p. 196; emphasis mine).

Kant’s famous and controversial conclusion is that space and time should not be conceived as external-world properties, but rather as internal structures that constrain possible perceptions—essentially stating that space and time are “idealistic” (Hatfield, 2006). But does PP actually posit that space and time are structures of perceptual systems rather than external real-world properties? Certainly many PP theorists will stop short of going this far “out there”. However, PP lends plenty of support to Kant’s conception of space and time. When taken together, the following two PP proposals—(1) perceptions* are* the predictions brains make about current sensory stimulation; and (2) spatial and temporal hyperpriors form and shape all perceptions of external objects—sound a lot like Kant’s claim that space and time are best thought of as originating, not from the “matter” of outer sensation, but rather from endogenous formal constraints on any perception of outer objects. While this potentially radical claim is not (yet) openly stated in current PP literature, some recent related work in cognitive science advances a similar line. For example, Hoffman and Prakash (2014, p. 20) conclude that “objects and space-time are simply species-specific perceptual adaptations” and Purves et al. (2015, p. 1) argue that our common assumption that perception delivers objective features of real-world properties should be replaced “with a paradigm in which perceptions reflect biological utility based on past experience rather than objective features of the environment”. This recent trend in neuroscience echoes Kant’s insistence that perception delivers only “appearances” and not “things-in-themselves,” as well as his doctrine that space and time are formal aspects of cognitive-perceptual systems and not objective features of external reality.

Kant considered his “forms of sensibility” so important that he proposed a new field of science to be devoted entirely to their investigation. “There must, therefore, be a science of all principles of* a priori* sensibility. I call such a science *transcendental aesthetic*” (Kant, 1996/1787, sec. A 20). Perhaps PP is answering Kant’s call for a science of *transcendental aesthetic*?

## A Note About The *A Priori*

At this point I would like to highlight an important *difference* between Kant’s accounts and those found in PP. Kant was primarily concerned with explaining *a priori* features of perception and cognition. He did not focus on the *empirical acquisition* of priors, he lacked evolutionary understanding, and he did not set out theories of learning. This creates a *prima facie* tension for comparisons between Kant and PP, since PP places emphasis on *learned* priors, “empirical Bayes”, and the idea that organisms perceive using probabilistic computations based on prior *experience*. However, this apparent tension might be dissolved if we keep in mind that PP is not a traditional “empiricist” theory, for it recognizes that many priors could be innate and biologically hard-wired (Clark, 2013), even if such wirings are ultimately the result of long-term phylogenetic experience. This conception of *a priori* seems to be in line with what Friedman describes as a “relativized and dynamical conception of* a priori* mathematical-physical principles, which change and develop… but which nevertheless retain the characteristically Kantian constitutive function of making the empirical natural knowledge thereby structured and framed by such knowledge possible” (Friedman, 2000, p. 370; see also Reichenbach, 1965). In a similar vein, Kitcher argues that what might *seem* to us to be *a priori* mathematical truth actually depend on “*the experiences of those who came before us in the mathematical tradition*” (Kitcher, 2000, p. 84).

Since Kant “uses “necessary” and “*a priori*” interchangeably” (Kitcher, 1980, p. 89; see also Kripke, 1972), we can compare Kant’s “necessary conditions” with the necessity of certain priors as outlined by PP without worrying about potential discrepancies that might arise from differing accounts regarding the exact nature and origin of *a priori* knowledge. Philosophers in general are far from certain about the relation between *a priori* and other notions, such as experience, innateness, nativism, rationalism, empiricism and so on (see Boghossian and Peacocke, 2000). Therefore, the fact that Kant and PP differ on their conception about the nature and origin of *a priori* structures in perceptual-cognitive systems does not preclude meaningful conceptual connections between Kant and PP, especially with regard to their accounts of the *functional role* of *a priori* principles.

## Generative Models and Kant’s Schemata

The problem of perceptual object recognition—how organisms are able to isolate meaningful objects from noisy and chaotic perceptual scenes—is a longstanding puzzle in philosophy as well as in the cognitive sciences. One way to study how biological brains recognize objects is to try to build *artificial* systems capable of object recognition and then look for the required design principles in brains (Griffiths et al., 2010). Early “connectionist” work in machine learning made important progress toward this effort, yet still “struggled to show appropriate representations in a deep multilayer context, and required large bodies of pre-classified data to power learning” (Clark, 2015b, p. 27). Operating largely on principles of weighted association and habit formation (fire together, wire together), these artificial systems proved unable to match the human ability to apply perceptual concepts and to recognize objects *in a general way*. “People learning new concepts can often generalize successfully from just a single example, yet machine learning algorithms typically require tens or hundreds of examples to perform with similar accuracy” (Lake et al., 2015, p. 1332).

To address this challenge, researchers in computer vision, machine learning and computational neuroscience have proposed that *generative models* might be central to solving the problem of perceptual object recognition and concept application (Dayan et al., 1995; Kersten et al., 2004; Friston, 2005; Hinton, 2007a,b; Clark, 2013). Generative models “capture the statistical structure of some set of observed inputs by inferring a causal matrix able to give rise to that very structure” (Clark, 2015a, p. 21). In other words, a system that uses generative models can estimate the causes of incoming sensations (and thus recognize objects) by *leveraging its own ability to produce similar sensations internally*. The key idea is that incoming sensory stimuli are “carved up”, not by comparing them to a database of previously encountered images, but rather by comparing them to more general endogenous “rules” (generative models) and then selecting (inferring) the model that is most likely able to generate the input patterns. In a hierarchical generative model, an upper layer is capable of producing—and thereby predicting—the activity patterns of the layer below. For perceptual systems, this means “that the model at layer N + 1 becomes capable of generating the sensory data (i.e., the input as it would there be represented at layer N (the layer below)) for itself” (Clark, 2015b, p. 26).

The generative model approach describes object recognition as a coordinated balance of both “top-down” and “bottom-up” flows of neural signals. The top-down signals instantiate a generative model—a matrix of possible causal structure—which “predicts” the causes of current sensations as it flows downward along the “backwards” or “feedback” anatomical neural pathways. Simultaneous with this top-down generation of predictions is a bottom-up neural signal flow against which the predictions are “matched” or “checked” (sometimes called a “recognition model”) and which flows along the “feedforward” neural connections (Kersten et al., 2004; Friston, 2005; Clark, 2013; Hohwy, 2013). There are many important details to this process, but the key point here is that theories of object recognition based on generative models involve *both* a top-down pass (endogenously generated from upper layers of the neural hierarchy) as well as a bottom-up pass (originating from lower layers of the neural hierarchy and ultimately from transduction at the external sense organ). The success of generative models in artificial perception, combined with the fact that the biological anatomy of brains boasts a neural architecture poised to support the types of connections required by generative models (Mumford, 1992; Friston, 2005; Yuille and Kersten, 2006; Clark, 2013), has motivated the proposal that brains recognize objects by way of a neurally-implemented top-down/bottom-up process involving generative models.

Crucial to the generative model solution to the problem of object perception is the “productive function” of the biological (or artificial) brain—its ability to endogenously generate sensory patterns. An artificial neural network based on generative models *develops its own* pattern-recognition abilities, not merely by habits of weighting and associating external stimuli, but by “dreaming”, using a “wake-sleep algorithm”, in which the system learns how to *generate* the patterns, *for itself*, by “imagining” different sorts of possible patterns “in fantasy”^3^. The knowledge of how to *generate* patterns is then used in order to *recognize* incoming patterns. “Here, instead of attempting to directly train a (synthetic) neural network to classify images, the network first learns to generate such images for itself” (Clark, 2015b, p. 27). Such a system then attempts to analyze and classify incoming stimuli, not by simply checking them against a database of previously-encountered images, but rather by identifying the endogenous rules or “imagination procedures” that it would use to generate the incoming stimuli for itself. This strategy provides a basis for achieving generalized perceptual concepts that are* less confined to particular token instances*, which has recently been demonstrated to match human performance on character recognition tasks (Lake et al., 2015). Hence Hinton’s (2007a) title “To Recognize Shapes, First Learn to Generate Images.”

Kant outlined a novel theory of perceptual object recognition and concept application^4^, which he called “schematism” (Kant, 1996/1787, sec. A 137). Kant’s schematism anticipates the generative model strategy in two major ways. Kant claims that: (1) object recognition requires a top-down generative process akin to imagination, in addition to a bottom-up sensory input flow and (2) that mind must classify perceptual objects, not by associating and comparing them to a set of previously encountered images, but rather by identifying the endogenous abstract rules it would use to generate the sensory patterns in imagination^5^.

Kant’s schematism arose from his dissatisfaction with the association-based habit-formation theories of object recognition of his contemporaries, much as generative models address the shortcomings of association-based connectionist approaches in machine learning and cognitive science. Kant argued that there were unacceptable limitations in the empiricist accounts of perceptual object recognition (primarily Hume and Locke) due in part to their appeal to laws of habit and association (Kant, 1996/1787; Hatfield, 1990). Kant does acknowledge that association and habit play an important role, which he called the “empirical laws of imagination,” grouped under the “*re*productive function of the imagination” (Kant, 1996/1787, sec. A 120–122; emphasis mine). Yet he objected to claims that such “reproductive” principles were sufficient to explain the *generalization* abilities of human perceptual object recognition. In objecting to this strategy, Kant accurately anticipates the “generalization problem” that has hampered connectionist approaches in machine learning. “No image whatever of a triangle would ever be adequate to the concept of a triangle as such. For it would never reach the concept’s universality that makes the concept hold for all triangles (whether right-angled or oblique-angled, etc.), but would always be limited to only part of this set” (Kant, 1996/1787, sec. A 141). Images of objects derived from experience—even complex clusters of associations of images—are *never* “adequate to the empirical concept.” Kant argued that, in order for perceptual concepts to take hold on incoming stimuli, a “third thing” must mediate the connection. “This mediating presentation must be pure… and yet must be both intellectual, on the one hand, and sensible, on the other hand. Such a presentation is the *transcendental schema*” (Kant, 1996/1787, sec. A 138). This statement anticipates the top-down/bottom-up interplay of the generative model strategy. Furthermore, like a generative model, “a schema is, in itself, always only a product of the imagination… a rule for the synthesis of imagination regarding pure shapes in space” (Kant, 1996/1787, sec. A 140–141). For Kant, evaluating perceptual stimuli using a “rule for synthesis” is the only way to avoid the generalization problem that comes with image association and matching strategies. “Images must always be connected with the concept only by means of the schema that they designate; in themselves the images are never completely congruent with the concept” (Kant, 1996/1787, sec. A 142).

In a recent demonstration of the power of the generative model paradigm, Lake et al. (2015) present an algorithm that can match human performance in a perceptual recognition task involving the identification of handwritten characters. In their approach, each handwritten character is represented in the system, not as an image of that character, but rather as “an abstract *schema* of parts, subparts and relations” (Lake et al., 2015, p. 1333; emphasis mine). This abstract *schema* takes the form of a mini-program, a set of instructions or *rules for generating images* of alphabet characters. “The model represents concepts as simple programs that best explain observed examples…” (Lake et al., 2015, p. 1332). To recognize incoming stimuli as alphabet characters, the system leverages its own capabilities for generating images, an approach they term “probabilistic program induction” (Lake et al., 2015, p. 1332).

This is very much in line with how Kant explained the role of schemata—a “concept always refers directly to the schema of imagination” (Kant, 1996/1787, sec. A 141)—though of course he did not conceptualize schemata as computer programs. However, Eco (2000) as well as Marconi (2003) both argue that Kant’s schemata can usefully be compared to procedural computer programs, and that they bear general resemblance to certain artificial intelligence strategies^6^. Moreover, Perlovsky et al. (2011) acknowledge and emphasize the Kantian roots of top-down model-based computational approaches to cognition and object recognition^7^. Kant’s schemata are the “third thing” that bridge concepts, on the one hand, with images, on the other, by being “homogenous” with both—they do this in virtue of their capacity as generic procedural rules for creating different types of structured sensory patterns (Kant, 1996/1787, sec. B 176). With his theory of schemata, Kant clearly anticipates a core part of the general strategy as found, for example, in the recent work of Lake et al. (2015). “In fact, it is schemata, not images of objects, that lie at the basis of our pure sensible concepts” (Kant, 1996/1787, sec. A 141).

**Figure 1:**
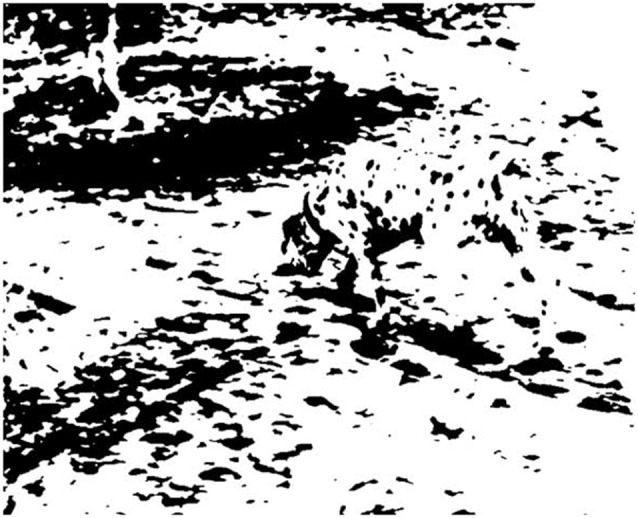
**First appeared in Gregory (1970)**.

Yuille and Kersten (2006), in one of the first articles to articulate a generative model strategy for problems in vision research, use a well-known example of an ambiguous black-and-white image (Figure 1), in which most people can, initially, see only random patches.

“Low-level cues for this image contain little evidence to activate a high-level dog model, and so naive subjects take a long time to detect the dog” (Yuille and Kersten, 2006, p. 302; see also Mumford, 1992) (The image contains a Dalmatian dog in a “drinking” pose). For many subjects, the dog becomes salient only after verbal prompts, after which it remains unavoidably salient. To explain this phenomenon, the authors advance the proposal that brains leverage top-down processing using generative models in order to achieve object recognition.

To explain how schemata are the key to picking out objects from within a noisy manifold of sensible intuitions, Kant uses his own dog example! “The concept dog specifies a rule whereby my imagination can trace the shape of such a four-footed animal in a general way, i.e., without being limited to any single and particular shape offered to me by experience, or even to all possible images that I can exhibit in concreto.” (Kant, 1996/1787, sec. A 141). Kant claims that schemata enable us to isolate an object from the sensory barrage and identify it as an animal which falls under the empirical concept *dog*^8^.

Thus, at least from the standpoint of a high-level comparison, generative models seem to fit Kant’s idea of schemata, from the general strategy right down to the illustrative examples. Clark even invokes the adverb “schematically” when explaining how a generative model “aims to capture the statistical structure of some set of observed inputs by tracking (one might say, by *schematically* recapitulating) the causal matrix responsible for that very structure” (Clark, 2013, p. 182; emphasis mine).

Kant cryptically comments that the full workings of schemata might forever remain mysterious to science. “The schematism of our understanding, i.e., its schematism regarding appearances and their mere form, is a secret art residing in the depths of the human soul, an art whose true stratagems we shall hardly ever divine from nature and lay bare before ourselves” (Kant, 1996/1787, sec. B 181). If I am on the right track linking Kant’s schemata to generative models, then perhaps PP reveals some of the “true stratagems” locked inside of the “secret art” of Kant’s notoriously mysterious schemata^9^.

## Analysis-By-Synthesis

Central to the secret art of generative models is a strategy known as “analysis-by-synthesis”. It is so-called because, as described above, the incoming sensations are *analyzed* by comparing them to the internal processes that could *synthesize* similar patterns endogenously. “We recognize objects and states of affairs, if these approaches are correct, by finding the most likely set of interacting factors (distal causes) whose combination would generate (hence predicts, and best accounts for) the incoming sensory data” (Clark, 2015a, p. 21). PP stresses that the generation of a structured scene *always* occurs with limited informational resources—information limited to that which is available from perspective of the organism (Clark’s “view from inside the black box”, or Hohwy’s “skull-bound brain”). Predictive brains leverage generative models to infer the “hidden causes” of the energetic stimulation occurring to the sense organs. “When the *combination* of such hidden causes (which span many spatial and temporal scales) settles into a coherent whole, the system has self-generated the sensory data using stored knowledge and perceives a meaningful, structured scene” (Clark, 2015a, p. 21; emphasis mine).

Kant placed great emphasis on a mental process that he called “synthesis” throughout *CPR*^10^. “By *synthesis*, in the most general sense of the term, I mean the act of putting various presentations with one another and of comprising their manifoldness in one cognition” (Kant, 1996/1787, sec. B 103). Kant introduces his technical definition of this term by connecting it at the outset with the activity of imagination and by stressing its crucial role in perception and cognition. “Synthesis as such, as we shall see hereafter, is the mere effect produced by the imagination… without which we would have no cognition whatsoever…” (Kant, 1996/1787, sec. A 78). Kant immediately contrasts synthesis with analysis and importantly, claims that: (1) synthesis is required for analysis; therefore (2) synthesis should be the primary target of any investigation about the fundamental workings of cognition.

Before any analysis of our presentations can take place, these presentations must first be given, and hence in terms of *content* no concepts can originate analytically. Rather, synthesis of a manifold (whether this manifold is given empirically or *a priori*) is what first gives rise to a cognition. Although this cognition may still be crude and confused at first and hence may require analysis, yet synthesis is what in fact gathers the elements for cognition and unites them to (form) a certain content. Hence if we want to make a judgment about the first origin of our cognition, then we must first direct our attention to synthesis (Kant, 1996/1787, sec. B 103).

Kant goes on to offer an elaborate account of three distinct stages of synthesis (“threefold synthesis”), along with a distinction between “pure” and “empirical” varieties of synthesis. I will not address such details here, however, because I am only arguing that Kant and PP share the fundamental proposal that *analysis* (the use concepts in sensation and cognition) proceeds by way of *synthesis* (the combining and ordering of sense data using what Kant calls the productive capacity of the imagination and its schemata).

## Imagination and Perception

Imagination plays a key role in the PP framework as it seems to be the engine that allows generative models to facilitate perceptions. Kant’s framework places imagination in a similar position.

Imagination is the power of presenting an object in intuition even without the objects being present. Now, all our intuition is sensible; and hence the imagination, because of the subjective condition under which alone it can give to the concepts of understanding a corresponding intuition, belongs to sensibility. Yet the synthesis of imagination is an exercise of spontaneity, which is determinative, rather than merely determinable, as is sense; hence this synthesis can* a priori* determine sense in terms of its form in accordance with the unity of apperception. To this extent, therefore, the imagination is a power of determining sensibility* a priori*; and its synthesis of intuitions in accordance with the categories must be the transcendental synthesis of imagination. This synthesis is an action of the understanding upon sensibility, and is the understanding’s first application (and at the same time the basis of all its other applications) to objects of the intuition that is possible for us (Kant, 1996/1787, sec. B152).

Kant argued tirelessly that imagination is the key to synthesis, and that synthesis lies at the basis of both perception and understanding. In discussing this aspect of Kant in the context of computational neuroscience Perlovsky et al. (2011, p. 86) state that “pattern recognition and artificial intelligence algorithms of recent past would not know how to relate to this.” In a footnote, Kant himself speculates on why the psychologists of his time did not recognize the key role of imagination in their accounts of perception.

That the imagination is a necessary ingredient of perception itself has, I suppose, never occurred to any psychologist. This is so partly because this power has been limited by psychologists to reproduction only, and partly because they believed that the senses not only supply us with impressions, but indeed also assemble these impressions and thus bring about images of objects. But this undoubtedly requires something more than our receptivity for impressions, viz., a function for their synthesis (Kant, 1996/1787, sec. A 120 n).

If Kant were writing today, he could not claim that the tight connection between imagination and perception has “never occurred to any psychologist,” because the psychologists who leverage PP-style theories are saying exactly this^11^. Dreams and mental imagery have played a role in PP theories since early formulations of top-down/bottom-up models of cortical activity (Mumford, 1992; Friston, 2005). For PP, imagination is the architecture by which generative models “generate” and produce predictive perceptions. This means that imagination is in some ways required for perception. “It means that perception (at least, as it occurs in creatures like us), is co-emergent with (something quite like) imagination” (Clark, 2015b, p. 26).

As we reflect on the tight kinship between PP and Kant, we might arrive at the following question. Why are Kant’s ideas seemingly reincarnated in contemporary PP theory? It is unlikely that the neuroscientists developing PP frameworks have drawn direct inspiration from deep readings in Kant’s *Critique of Pure Reason*. So are the links I have presented here the result of mere coincidence? Indirect influence? Or can they be traced directly to Kant through the influence of Helmholtz?

## Predictive Processing, Helmholtz and Kant

The current PP paradigm emerged from early work on generative models, and this early work explicitly identifies itself as being directly inspired by the work of 19th century German scientist Hermann von Helmholtz (1821–1894). For example, the seminal article on the use of generative models in machine perception, titled “The Helmholtz Machine,” states that “Following Helmholtz, we view the human perceptual system as a statistical inference engine whose function is to infer the probable causes of sensory input” (Dayan et al., 1995, p. 889). Homage to Helmholtz is also given in early proposals of vision as Bayesian inference, where “vision is treated as an inverse inference problem, in the spirit of Helmholtz, where the goal is to estimate the factors that have generated the image” (Yuille and Kersten, 2006, p. 301). The “free energy principle”—a far-reaching PP model of the entire nervous system developed by Friston et al. (2006)—is introduced with the claim that “if one formulates Helmholtz’s ideas about perception in terms of modern-day theories one arrives at a model of perceptual inference and learning that can explain a remarkable range of neurobiological facts” (Friston and Stephan, 2007, p. 417). More recent overviews of PP also identify Helmholtz as the ancestral precedent of the overall PP paradigm (Bubic et al., 2010; Friston, 2012; Clark, 2013; Hohwy, 2013).

Core PP ideas pioneered by Helmholtz include the central idea that percepts are akin to (mostly unconscious) “inferences”, the notion that perception might involve a process analogous to scientific induction, and the understanding of illusions as “optimal percepts” that are generated based on the most likely causes of sensations (Helmholtz, 1925). Helmholtz also tackled the issue of what is now termed “top-down” cognitive influence on perception, stating that “we cannot altogether avoid speaking of the mental processes that are active in the sense-perceptions if we wish to see clearly the connection between the phenomena and to arrange the facts in their proper relation to one another” (Helmholtz, 1925, p. 2). Helmholtz then immediately states that *Kant* articulated the proper relation between mental processes and perceptual processes. “The keenest thinkers, philosophers like Kant for instance, have long ago analyzed these relations correctly and demonstrated them…” (Helmholtz, 1925, p. 2).

This respectful reference to philosophy—and to Kant in particular—is a consistent theme in Helmholtz. He took philosophy very seriously and published several philosophical works over the course of his scientific career, throughout which there is “frequent insistence on his Kantian ancestry” (Hatfield, 1990, p. 169). Helmholtz declared himself to be a “faithful Kantian,” and believed that he was providing a scientific implementation and “operationalization” of Kant’s epistemology and (anti-) metaphysics (Hatfield, 1990; Lenoir, 2006). Helmholtz saw his work as remaining true to Kant’s epistemological project, and even explained how his scientific theories of perception provided important revisions to certain conclusions made by Kant (Hatfield, 1990; Lenoir, 2006). “Helmholtz thus considered himself more consistently Kantian than Kant had been himself” (Lenoir, 2006, p. 141).

If we consider the fact that PP is widely seen as a contemporary formulation of the work of Helmholtz, taken together with the fact that Helmholtz embraced Kant’s philosophy, the links between PP and Kant should not come as a surprise. In this regard, PP can even be seen as a major step in the evolution of Kant’s transcendental psychology. Helmholtz brought the ideas of Kant to the table as he developed scientific theories of the psychology and physiology of perception, and the PP paradigm is now bringing the theories of Helmholtz—and thus the ideas of Kant—into contemporary neuroscience and machine learning research. The links between Kant and PP that I defend in this article seem much less mysterious when we keep in mind the fact that PP is part of a Kantian lineage inherited directly from Helmholtz.

## Conclusions

Prompted by Hume’s questioning of the origin of causal structure in percepts, Kant applied his top-down analytical method to reverse-engineer cognition and perception. From the start of his endeavor, Kant maintained that, if we are to make any progress, we must first invert the traditional account of the relation between the structure of cognition and the objects presented in outer perception, so that we “instead assume that objects must conform to our cognition” (Kant, 1996/1787, sec. B xvi). Proceeding from this initial premise, Kant then developed an elaborate model of perception and cognition that proposed many novel concepts and specific theoretical components. The components of Kant’s system discussed in the present treatment include the proposal that space and time are formal structures of perceptual-cognitive processes necessary for outer perception; that external perception and object recognition is made possible by chains of endogenous procedural rules capable of generating mental imagery; that cognitive-perceptual understanding proceeds according to alternating iterative steps of analysis and synthesis; and that “imagination is a necessary ingredient of perception itself” (Kant, 1996/1787, sec. A 120 n).

As I have shown in this article, PP proposes an account of perception and cognition that echoes these core aspects of Kant in specific ways. This, of course, is not to say that all of PP is “Kantian” or in complete agreement with Kant’s entire transcendental philosophy. PP’s probabilistic and evolutionary approach (not to mention its computational and neuroscientific underpinnings) goes beyond Kant’s insights in ways that Kant could not have imagined. Indeed, operationalizing Kant was Helmholtz’s explicit intent for his work on perception, much like the intent of PP has been to enhance the insights of Helmholtz with a modern neurocomputational and probabilistic toolset.

All of this comparison to Kant prompts an important question: Is it even possible to arrive at a formulation of PP that avoids these Kantian aspects? Or does PP by its very nature entail a Kant-style conception of perception, cognition, and their relation to the external world?

It is my hope that the links between PP and Kant defended in this article will persuade PP theorists that Kant’s work is directly relevant to the historical context of PP, and perhaps even hint that further important insights might await those who embark on PP-savvy readings of Kant. Some cognitive scientists already attest to the usefulness of Kant’s ideas within neuroscience and artificial systems research (Marconi, 2003; Perlovsky et al., 2011; Fazelpour and Thompson, 2015). Others will object that dabbling in the metaphysics of long-gone philosophers (especially Kant) is not a wise way to move forward within neuroscience. However, here I am in agreement with Edelman (2012, p. 3), who states that philosophy—especially history of philosophy—has an important role to play in “sharpening psychology’s theoretical tools by focusing on its conceptual foundations in a broad perspective, which includes philosophical considerations and indeed, metaphysics…”.

Kant scholars might likewise benefit from PP-informed readings of Kant. Clark argues that PP represents “a genuine departure from many of our previous ways of thinking about perception, cognition, and the human cognitive architecture” (Clark, 2013, p. 187). The historical links presented in this article might call Clark’s claim into question. However, I would argue that, in spite of being anticipated by Kant, PP nonetheless represents a “genuine departure” as Clark words it, because Kant’s work on perception and cognition never really caught on within psychology. Kant’s psychology, which has been called “the dark side of the critique,” has been deemed shameful by many 20th century analytic philosophers (Kitcher, 1993, p. 3). Many attempts have been made to “salvage” Kant’s “austere” ideas from the “incomprehensible” arguments of his transcendental psychology (Bennett, 1966; Strawson, 1966; Wolff, 1970; Guyer, 1987; Allison, 2004). “Powerful currents within and without Kant scholarship have combined to keep transcendental psychology out of the mainstream, beyond the pale of serious philosophical discussion” (Kitcher, 1993, p. 5). Much of cognitive science has reflected this aversion to Kant-style frameworks, readily evidenced by the preponderance of bottom-up, feedforward models of brain activity. “Neuroscientific studies of structural and functional brain connectivity in the past two decades, however, provide strong support for a view of the mind much closer to that which Kant envisioned” (Fazelpour and Thompson, 2015). If PP proves to be an important advance for cognitive science, and if the links to Kant discussed here hold up, then this will support the perspective of Kitcher and Hatfield that “it is not crazy to take Kant’s psychology seriously” (Kitcher, 1993, p. vii).

## Author Contributions

LRS researched and wrote the article.

## Conflict of Interest Statement

The author declares that the research was conducted in the absence of any commercial or financial relationships that could be construed as a potential conflict of interest.

## Footnotes

^1^However, PP should not be conflated with broader and more general Bayesian approaches to cognition (Griffiths et al., 2008; Blokpoel et al., 2012; Friston, 2012).

^2^Clark doesn’t explain further, which is in part what motivated the present article.

^3^Clark (2015a) reports a handful of such references to “imagination” in generative model-based computer vision and machine learning techniques.

^4^Kant proposed that both “empirical concepts” (perceptual/sensory concepts) as well as “pure concepts” (abstract cognition) required schemata.

^5^Perlovsky et al. (2011) present their own computational model of concepts and object recognition, which they explicitly identify with Kant on similar grounds.

^6^Neither Eco nor Marconi seem to have been aware of the idea of a generative model when they compared Kant’s schemata to computer procedures.

^7^Perlovsky et al. (2011) do not discuss Kant’s schematism in comparison with computational approaches.

^8^For more fun discussion of imagination, perception and recognizing dogs, see Strawson (1970) and Eco (2000).

^9^In his article “Why cognitive science needs philosophy and vice versa, philosopher of science Paul Thagard argues that cognitive science does need philosophy, “but it does not need most styles of philosophy… although there may be occasional theoretical ideas such as *Kant’s theory of schemas* and Frege’s theory of relations that prove scientifically useful” (Thagard, 2009, p. 249; emphasis mine).

^10^We should exercise caution when discussing Kant’s use of the term “synthesis”, as he used it in (at least) two distinct ways. See Kitcher (1996, p. xliv) for important clarifications.

^11^The central role of imagination is also recognized by computational neuroscientists outside of PP, such as Perlovski, who emphasizes that his theories of dynamic logic and neural modeling fields, as well as Carpenter and Grossberg’s (1999) adaptive resonance model “both describe imagination as an inseparable part of thinking”. (Perlovsky et al., 2011, p. 86).
